# Suppression of reproductive behaviour and gonadal function in female horses—An update

**DOI:** 10.1111/rda.14129

**Published:** 2022-05-01

**Authors:** Christine Aurich, Martim Kaps

**Affiliations:** ^1^ Artificial Insemination and Embryo Transfer Department for Small Animals and Horses Vetmeduni Vienna Vienna Austria

**Keywords:** behaviour, oestrus suppression, female horse, ovulation

## Abstract

The behaviour of mares is often detrimental to their performance resulting in frequent demand for methods to suppress gonadal function. In addition, prevention of unintended reproduction especially in feral horse populations may require methods for suppression of gonadal function. Surgical ovariectomy is a safe method but not an acceptable approach in feral mares and undesired in mares where future breeding is considered. There are different approaches for artificial prolongation of the luteal phase resulting in transient inhibition of oestrus and ovulation. Among those, treatment with natural or synthetic progestogens is considered the most common and successful method. Whereas application of intrauterine devices may result in prolongation of luteal function in non‐pregnant mares, intrauterine insertion of glass balls is no longer recommended because of complications in individual mares. There are several safer alternatives that may be of interest, especially for population control in free‐roaming horses. Treatment with long‐acting deslorelin implants inhibited ovulation and oestrus behaviour in mares for limited and variable time intervals in a dose‐dependent manner. The effect of GnRH vaccines varies considerably among individual mares, is age dependent, and oestrus‐like behaviour may still occur. Contraception via immunization against native porcine or recombinant zona pellucida antigen is successful, but immunocontraception is as much a result of ovarian inactivity as an antibody‐based block to sperm‐oocyte binding. In conclusion, several treatments for suppression of gonadal function in mares are available, but there are advantages and disadvantages associated that have to be considered. The treatment of choice will thus differ with regard to the demands.

## 1 INTRODUCTION

In female horses, oestrus behaviour is often associated with a moody temperament and undesired behaviour with regard to their use in equestrian activities (Pryor & Tibary, 2005). The behaviour of mares is therefore often detrimental to their performance (Hedberg, Dalin, Forsberg, Lundeheim, Hoffmann, et al., 2007; Stout & Colenbrander, 2004). Trainers, riders and persons handling mares frequently complain about such problems and demand for methods to suppress gonadal function with the aim to reduce oestrus behaviour and the associated problems. Whereas such treatment of healthy mares may stimulate controversial discussions, it is certainly justified when in individual mares, excessive and even aggressive behaviour occurs during oestrus. Suppression of ovarian function should also be considered in mares that are not intended for breeding but suffer from abdominal discomfort caused by large pre‐ovulatory follicles and ovulation (Cox & DeBowes, 1987; Hooper et al., 1993; McCue, 2003). Finally, prevention of unintended reproduction either in individual mares or with the aim to avoid overpopulation may require methods for suppression of gonadal function (Joonè et al., 2019; Swegen & Aitken, 2016), but there are different demands with regard to efficiency and controllability of such treatments when used for population control or management of individual fertility, respectively (Table 1). While in feral horses contraception is the major aim, changes in reproductive behaviour should be avoided allowing for maintenance of physiological social structures. In addition, limitations in the accessibility of feral horses require long‐acting and easily applicable procedures available at low costs (Hobbs & Hinds, 2018). In contrast, in individual sport horse mares, suppression of sexual behaviour is the major aim. A transient loss in fertility may be tolerable but in most cases the owner will desire to breed the mare in the future. While cost‐effective solutions may be preferable, effectiveness is more important.

**TABLE 1:** Information on efficacy of treatment, expected fertility after cessation of treatment and usability for either population control in wildlife horses or suppression of oestrus behaviour in individual mares

Approach	Efficacy	Fertility after cessation of treatment	Population control in wildlife horses	Suppression of oestrus behaviour in individual mares
Bilateral ovariectomy	Unlimited	Not applicable	**‐‐**	+
Induction of luteal tissue persistence	Transient	Unchanged	**‐‐**	+
Altrenogest (oral administration)	Transient	Unchanged	‐‐	++
Intrauterine devices	Transient	May be reduced	++	+
GnRH‐agonist slow‐release implants	Transient	Unchanged	‐‐	+
Anti‐GnRH vaccination	Transient, but future ovarian function may be impaired	May be reduced	++	++
Vaccination against ZP antibodies	Transient, but future ovarian function may be impaired	May be reduced	++	+

Abbreviations: ‐‐, not advisable; +, may be considered; ++, recommended treatment.

**FIGURE 1:**
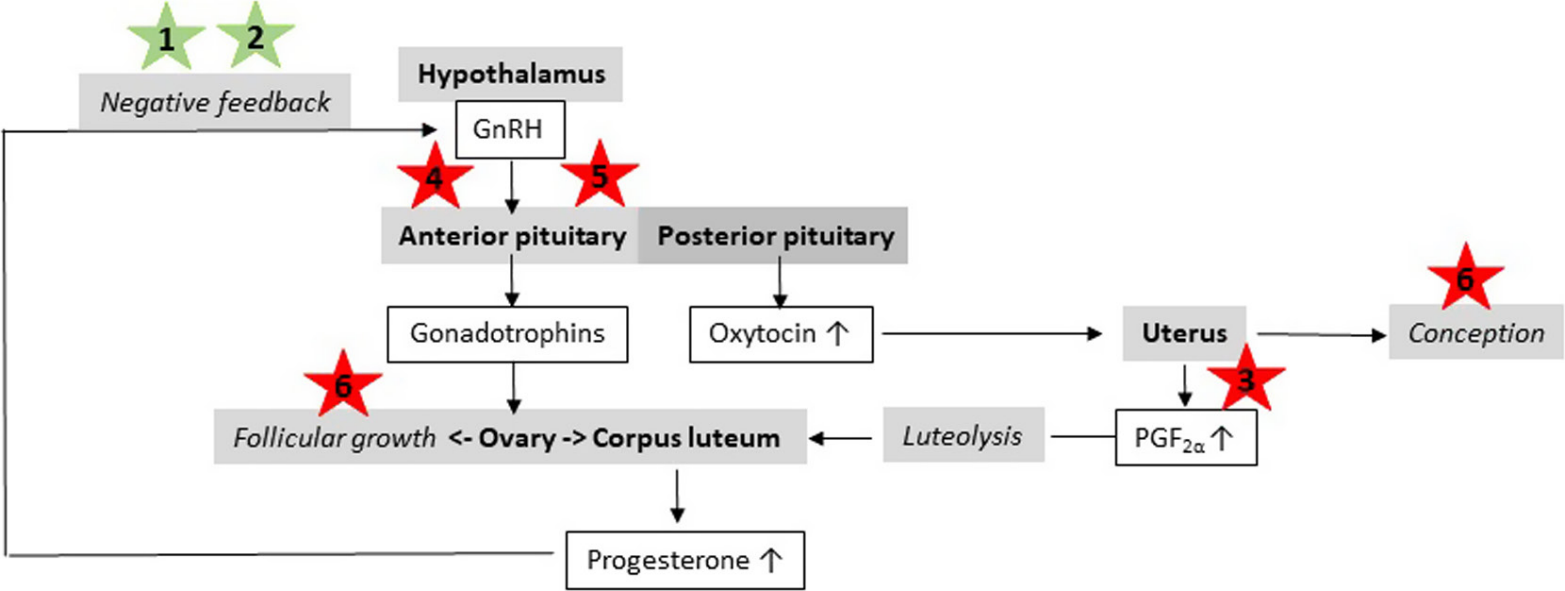
Regulation of the oestrous cycle in female horses and targets for inhibition of oestrus: 1. prolongation of the luteal phase, 2. treatment with progestins, 3. application of intrauterine devices, 4. treatment with long‐acting GnRH analogues, 5. immunization against GnRH, 6. immunization against zona pellucida proteins. In green: prolongation/enhancement of the physiological mechanism; In red: inhibition/disruption of the physiological mechanism

Although surgical removal of the ovaries in female horses via laparoscopy is a safe method (Röcken et al., 2011), it may not be an acceptable approach in feral mares and undesired in mares where future breeding is considered. What is even more, in ovariectomized mares, oestrous behaviour may still occur because of adrenal oestradiol synthesis (Asa, 1986; Hedberg, Dalin, Forsberg, Lundeheim, Sandh, et al., 2007). Ovariectomized mares can thus display prolonged periods of oestrus‐like behaviour (Asa, 1980; Hooper et al., 1993; Palmer, 1993; Roessner et al., 2015). This complication clearly questions ovariectomy as the best option for treatment of undesired mare behaviour. Fortunately, there is a wide range of methods for suppression of either oestrous behaviour or ovarian function in female animals (Figure 1), but not all of them will result in satisfying results in the female horse. The present review aims at introducing these methods together with their limitations and future perspectives for their improvement as well as alternative application in mares.

## 2 INTERRUPTION OF THE HYPOTHALAMIC–PITUITARY–GONADAL AXIS

Supplementation of exogenous steroid hormones to an individual will usually suppress GnRH as well as subsequent LH and FSH release by activating the negative feedback mechanism of the hypothalamic–pituitary–gonadal axis (female horse: Aurich et al., 1995; Garcia et al., 1979; Garcia & Ginther, 1978). Supplementation of exogenous hormones can therefore suppress fertility. Alternatively, a sustained exposure to GnRH, its analogues or antagonists may reduce stimulation of gonadotrophin synthesis and/or secretion by down‐regulation of GnRH receptors. Both approaches thus result in interruption of the hypothalamic–pituitary–gonadal axis. Because these mechanisms are reversible, they will transiently inhibit gonadal function. There are, however, considerable differences with regard to their effectiveness depending on the species and sex.

### 2.1 Artificial prolongation of the luteal phase

One possible approach for suppression of oestrus and ovulation in mares is an artificial prolongation of the luteal phase. This condition may occur spontaneously and result in persistence of luteal tissue for 2–3 months (Allen & Rossdale, 1973; Ginther, 1990; Newcombe, 1997; Stabenfeldt et al., 1974). In non‐pregnant mares, this phenomenon is associated with dioestrus ovulations in at least part of the affected horses (Newcombe et al., 1997). If a dioestrous ovulation occurs after day 9 of the cycle (day 0 = day of ovulation), there will be a prolonged luteal activity because at the time of endogenous luteolysis (i.e. day 14) luteal tissue from the dioestrous ovulation is still irresponsive to the luteolytic signal although the primary corpus luteum may undergo luteolysis (Ginther, 1990; Newcombe, 1997). Induction of ovulation with 3,000 I.U. human chorionic gonadotrophin (hCG) in luteal phase mares with an ovarian follicle >30 mm in diameter resulted in ovulation in 3 of 4 mares within 72 hr and prolonged the luteal phase for 58–82 days (Hedberg et al., 2006). In contrast, treatment with the GnRH‐agonist deslorelin failed to induce luteinization or ovulation of dioestrous follicles in another study. The difference in the effectiveness between hCG and deslorelin may be associated with a reduced availability of pituitary LH in dioestrus mares (Glazar et al., 2004).

Alternatively, pharmacologic inhibition of endogenous luteolysis will extend corpus luteum lifespan. Treatment of non‐pregnant mares with oxytocin in large doses during mid‐dioestrus (60 I.U. once daily on days 7–14 after ovulation) inhibited the endometrial secretion of prostaglandin F_2α_ (PGF_2α_; Keith et al., 2013). Spontaneous luteolysis was subsequently delayed until approximately day 70 after ovulation in 60–70% of treated mares (Vanderwall et al., 2012, 2015). The protocol requires determination of the day of ovulation to allow for precise timing of oxytocin treatment. Extension of the duration of oxytocin treatment to 29 days, however, allowed initiation of treatment randomly at any time in the oestrous cycle with no loss in efficacy (Parkinson et al., 2020; Vanderwall et al., 2016), but was even more labour‐intensive.

Removal of the conceptus after maternal recognition of pregnancy, that is after day 15 after ovulation, results in prolongation of the luteal phase in mares because by this time, the luteolytic mechanism has been suppressed and presence of the conceptus is now longer required to maintain corpus luteum lifespan (Hershman & Douglas, 1979). Termination of pregnancy between days 16 and 20 after ovulation by manual ablation (crushing) of the conceptus extended luteal function and inhibited oestrus‐related behaviour until approximately day 82 after ovulation (Lefranc & Allen, 2004). The treatment was highly successful, and none of the mares returned to oestrus after conceptus crushing. This approach requires, however, successful breeding; it may therefore be a challenge in mares with fertility problems and raises ethical concerns.

### 2.2 Application of intrauterine devices

Application of intrauterine devices may result in prolongation of luteal function in non‐pregnant mares. Intrauterine insertion of glass balls (diameter 25–35 mm) shortly before or after ovulation proved successful in inducing prolonged luteal function in approximately 40% of treated mares with a mean duration of the luteal phase of 90 days (Nie et al., 2003). While the 25 mm glass balls were expelled in 50% of mares, the larger balls were all maintained. The treatment initially became quite popular for suppression of ovarian function and oestrous behaviour in mares. In the field, however, efficiency was often low (e.g. Argo & Turnbull, 2010) and—more importantly—numerous complications were reported with several being caused by fragmentation of glass marbles and shards becoming adhered to the uterine wall (de Amorim et al., 2016; Freeman & Lyle, 2015; Katila, 2015; Morris et al., 2017; Turner et al., 2015). The approach can thus no longer be recommended (Vanderwall, 2015). Water‐filled plastic balls (20 mm diameter) have been reported a safer alternative with similar effectiveness (Rivera del Alamo et al., 2008). More recently, a frameless, self‐assembling device (iUpod) made of shatter‐proof inert material was developed for intrauterine administration in mares (Gradil et al., 2019). The device is reported to be easy to administer and to retrieve. The device was highly effective with regard to oestrus behaviour suppression and had high retention rates. Rideability of mares was reported to be improved (Gradil et al., 2019).

Although the mechanism of action of such intrauterine devices is not completely elucidated, the initial hypothesis that they might simulate the presence of a conceptus and thus prolong the luteal phase (Nie et al., 2003; Rivera del Alamo et al., 2008) can no longer be supported. In contrast, it is highly likely that presence of a foreign intrauterine object is associated with inadequate endometrial prostaglandin release (Nie et al., 2003). This hypothesis was recently supported by the finding of a modified protein composition of the endometrial fluid of mares treated with intrauterine water‐filled plastic balls (Rivera del Alamo et al., 2021).

Intrauterine devices are also an interesting approach for population control in free‐roaming horses. They are likely to fulfil the requirements of a long‐lasting contraceptive that does not require the animal being recaptured frequently (Holyoak et al., 2021). In this context, the sexual behaviour of horses living under wildlife conditions has to be considered. The structure of harem bands depends on the social bond among the harem stallion and his mares. Mating will take place irrespective of the mares' reproductive status (Crowell‐Davis et al., 2007). Breeding mares fitted with an intrauterine device should neither induce its loss nor damage of the male and female genital organs. Already in 1995, there was a report on effective, safe and practical contraception in mares kept together with stallions in paddocks by intrauterine introduction of O‐ring‐shaped silastic devices (Daels & Hughes, 1995). Similar success was achieved when iUpods (Gradil et al., 2019; Hoopes et al., 2021) or Y‐shaped silastic devices (Holyoak et al., 2021) were used. In this study, there was a remarkably lower retention rate when O‐ring shaped silastic devices were used (Lyman et al., 2021). When mares were kept with a stallion, a considerable percentage of mares showed oestrous behaviour (Lyman et al., 2021) and developed signs of endometritis (Daels & Hughes, 1995; Hoopes et al., 2021). Nevertheless, fertility was not considerably affected when intrauterine devices were removed and mares allowed mating (Daels & Hughes, 1995; Holyoak et al., 2021; Hoopes et al., 2021). The contraceptive effects of devices that do not break or hurt the endometrium are thus at least in part reversible. These results demonstrate that intrauterine devices are successful for contraception but do not necessarily suppress oestrous behaviour of mares. They can therefore be considered safe and effective with regard to population control in free‐roaming horses. Their use may, however, not necessarily result in the desired improvement of the handleability of sport horse mares. Regardless of the purpose, development of endometritis is possible, and although the condition is not detrimental to the mares' general health, this may raise concerns.

### 2.3 Treatment with progestogens and progestins

Instead of prolongation of corpus luteum function, treatment with natural or synthetic progestogens suppresses gonadal function in mares. Injection of progesterone in either a dose of 100 mg daily or 400 mg every other day reliably suppressed oestrus behaviour in mares (Loy & Swan, 1966). Treatment with progesterone requires frequent injections and reactions at the injection side may occur. In addition, progesterone for injection is not available in many countries. In consequence, the approach is not common. In contrast, because of the availability of altrenogest for oral application and the proven effectiveness in the horse (McKinnon et al., 2000), daily long‐term oral application of altrenogest is the most common treatment for suppression of ovarian function in mares (McCue et al., 2016; Vanderwall, 2015). It has been successfully used for decades (Squires et al., 1979; Lofstedt & Patel, 1989). When treatment is declared, altrenogest is not classified as doping or prohibited medication for mares in equestrian sports (International Equestrian Federation, 2022). In horse racing, treatment with altrenogest on race days is prohibited (International Federation of Horseracing Authorities, 2021). In general, treatment with altrenogest is no longer recommended because of a possible contamination with the anabolic agents, trendione and trenbolone (British Horserace Authority, 2018). No adverse effects of altrenogest treatment on fertility have been described (Squires et al., 1983), but an increased susceptibility for uterine inflammation has been suggested (Burger et al., 2008; Fedorka et al., 2019), especially after prolonged periods of continuous treatment. After cessation of daily treatment during the breeding season, mares will return to oestrus within 5 days (Squires et al., 1983). When altrenogest treatment is started late in dioestrus, however, there is a high incidence of ovulations during treatment and the subsequent luteal phase is frequently prolonged because of failure of corpus luteum regression. Mares may therefore fail to come into oestrus after cessation of treatment (Daels et al., 1996). Concerns with regard to safety of the drug for humans handling the horses have been raised because of reports of adverse effects after skin contact with the substance (U.S. Food & Drug Administration, 2019) and should be taken into account. Injectable sustained‐release formulations of altrenogest are therefore beneficial with regard to safety and labour intensity. Injectable formulations of altrenogest effectively suppress oestrus for 72 hr (Storer et al., 2009) or even 148 hr (McConaghy et al., 2016); they are available for example in the US, but currently not in Europe.

There are reports that progestogens and their metabolites may exert sedative effects (Majewska et al., 1986). In female rats, antidepressant and anxiolytic effects were described (Laconi et al., 2001; Martinez‐Mota et al., 1999; Picazo & Fernandez‐Guasti, 1995). We have recently demonstrated that altrenogest reduced the stress response of 3‐year‐old mares during their initial equestrian training (Kaps et al., 2022). Beneficial effects on the rideability of mares exposed to continuous altrenogest treatment may therefore not only depend on the inhibition of oestrus but also on modulatory effects on the central nervous system. These findings might stimulate discussions if the permission of altrenogest treatment in mares taking part in FEI (International Equestrian Federation) competitions requires reconsideration.

### 2.4 Treatment with long‐acting GnRH analogues

Slow‐release implants containing the GnRH analogue deslorelin are licensed in Europe for the induction of transient infertility in adult male dogs and ferrets (EMA 2012–06–05) and have been commercialized (Suprelorin, Virbac). There are several advantages such as ease of use, biocompatibility and safety. In the horse, the effectiveness of GnRH receptor down‐regulation was previously questioned. Because of irregularities in the oestrous cycle of mares previously treated with 2.1 mg deslorelin implants for ovulation induction, the effectiveness of these implants to down‐regulate GnRH receptors was eventually proven (Farquhar et al., 2001, 2002; Johnson et al., 2000; McCue et al., 2002). In stallions, however, treatment with either one 4.7 or two 9.4 mg deslorelin implants did neither inhibit sexual behaviour nor testicular function although the pituitary responsiveness was clearly suppressed after treatment (Gautier et al., 2018). In contrast, treatment of cyclic mares of different breeds did not only result in a transient down‐regulation of pituitary GnRH receptors but was also associated with inhibition of ovulation and oestrus behaviour. The effect was dose‐related with spontaneous ovulation being delayed on average 40 days in Shetland mares treated with one 9.4 mg deslorelin implant, 20 days in Shetland mares treated with one 4.7 mg deslorelin implant (Kaps, Okada, Gautier, Aurich, Scarlet, et al., 2021) and <10 days in Haflinger mares treated with one 9.4 mg implant (Kaps, et al., 2021). Slow‐release deslorelin implants are thus not a reliable tool for long‐term suppression of ovarian function in horses, but may be worth considering when short time effects are desired.

Although ovarian activity was only suppressed for a limited time in Haflinger mares, changes in follicular development were demonstrated. The treatment increased the subpopulation of follicles 10–15 mm diameter in size and raised AMH concentrations in plasma. These results suggest a prolonged modulation of gonadotrophin secretion after deslorelin implant application stimulating the growth of small antral follicles. Because in equine assisted reproduction, the number of follicles available for aspiration by ultrasound‐guided ovum pick up (OPU) is a major limiting factor (Cuervo‐Arango et al., 2019) pretreatment of mares with deslorelin implants might be considered an approach for improving the oocyte yield.

### 2.5 Immunization against GnRH

Immunocastration vaccines have been developed with the aim to limit fertility in feral, livestock and companion animals (Bradley et al., 1999). Induction of anti‐GnRH antibodies with GnRH vaccines prevents binding of endogenous GnRH to its pituitary receptor. Because GnRH itself is small and therefore poorly immunogenic, the molecule is conjugated to a foreign protein (e.g. ovalbumin) and combined with an adjuvant. Early studies used low concentrations of GnRH conjugates and elaborate immunization protocols (e.g. Garza et al., 1986) that were efficient but not usable under field conditions. Eventually, the GnRH vaccine, Equity (Zoetis), was commercially available for oestrus control in wild roaming horses in Australia and New Zealand, but the production has been discontinued. The vaccine Improvac (Zoetis) was developed for immunocastration of male pigs and is approved in more than 60 countries. The vaccines differ with regard to their adjuvant but are both effective in horses (Imboden et al., 2006). There was no difference with regard to the efficacy of the vaccine when either a 200 or 400 mg dose was injected to 2‐year‐old fillies (Tshewang et al., 1997). Suppression of ovarian function in mares occurs within 4 weeks after the second GnRH vaccination, and booster injections are recommended at 6‐month intervals. The effect of vaccination on ovarian function, however, varies considerably among individuals and lasts from 28 to more than 100 weeks. There was no clear correlation with GnRH antibody titres (Elhay et al., 2007; Imboden et al., 2006; Schulman et al., 2013), but there was a considerable age effect with regard to the interval to resumption of cyclic ovarian activity after GnRH vaccination that was considerably longer in younger (≤4‐year‐old) mares (Schulman et al., 2013). Mares vaccinated at an early age may therefore remain infertile for several years. Although ovarian function is inhibited, oestrus‐like behaviour may occur in GnRH‐vaccinated mares (Dalin et al., 2002; Imboden et al., 2006). Adverse reactions to the injection have been reported and range from swelling and pain at the injection site, stiffness of the neck to pyrexia and apathy (Imboden et al., 2006), whereas others classified the reactions as mild and transient (Elhay et al., 2007; Schulman et al., 2013). Nevertheless, awareness of the owner to possible adverse effects is necessary and proactive treatment with non‐steroidal anti‐inflammatory drugs for some days should be considered. In conclusion, GnRH vaccination is a valuable tool for suppression of oestrus behaviour and ovulation in sport horse mares if future use as a broodmare is not of importance. The treatment may also be considered a method to determine whether surgical ovariectomy could be effective in overcoming behavioural problems in a mare. In competing mares, anti‐doping and controlled medication regulations have to be taken into account. The treatment is prohibited in FEI competitions (International Equestrian Federation, 2022) and in horse racing (International Federation of Horserace Authorities, 2021).

## 3 IMMUNIZATION AGAINST ZONA PELLUCIDA ANTIGENS

Contraception by immunization against porcine zona pellucida (pZP) antigen has been applied to various species including horses (Kirkpatrick et al., 2009). The suggested mechanism of action of anti‐pZP vaccines was an antibody‐based inhibition of sperm‐oocyte binding and fertilization (Barber & Fayrer‐Hosken, 2000). This was considered a benefit because no influence on ovarian function and ovulation of treated females was expected and therefore contraceptive treatment would not interfere with the social structure of free‐ranging horses (Fayrer‐Hosken et al., 2008). The horse was the first species where a native pZP vaccination has shown to be effective for inhibition of fertility (Liu et al., 1989). Vaccination of 14 mares for four times at 2–4 week intervals prevented pregnancy in 12 of them within the subsequent 7 months. After antibodies decreased, conception occurred suggesting that immunization resulted in transient infertility. Histology did not reveal major changes in domestic mares' ovaries (Liu et al., 1989). Several studies with different vaccination schedules and vaccine formulations followed presenting confirmative data (reviewed by Joonè et al., 2019). Because in several species, ovarian dysfunction after pZP immunization was reported, effects on ovarian function and histology as well as immunological responses and safety in female horses were revisited more recently (Joonè, Bertschinger, et al., 2017; and Joonè, et al., 2017). Results from a study in seven pony mares suggest that infertility during pZP immunocontraception was, at least, as much a result of ovarian inactivity as it was caused by an antibody‐based block to sperm‐oocyte binding (Joonè et al., 2019). A vaccine incorporating recombinant (re) ZP proteins included into this study resulted only in partial contraceptive effects and lower antibody titres (Joonè, Bertschinger, et al., 2017). Subsequent modification of the reZP vaccine, that is formulation with non‐Freund's adjuvants, induced the development of anti‐ZP antibody titres comparable to those in native pZP‐treated horses with only minor side effects (Nolan et al., 2019). There were also varying degrees of ovarian suppression with a higher percentage of mares ceasing cyclic ovarian activity after reZP‐only immunization in comparison to pZP or pZP‐reZP combined vaccination. Changes in ovarian function are most probably related to a cell‐related immune response to ZP‐based vaccines (Joonè et al., 2019). Antibody titres were a significant predictor of ovarian inactivity (Nolan et al., 2018).

## 4 CONCLUSIONS

Although several treatments for suppression of gonadal function in mares are available, they differ with regard to their reliability, effectiveness, practicability, safety for humans and animals as well as ethical aspects. Advantages and disadvantages thus have to be considered and the treatment of choice will differ with regard to the demands. Suppression of oestrus in an individual sport horse mare or in a population of feral mares will require different approaches and pose different problems. The use of vaccines for contraception in feral horses has several limitations because treated horses require individual identification and retrieval to allow for maintenance of the vaccination schedule, long‐acting formulations must be hand‐injected, and learned aversion in treated horses may challenge repeat treatments (Joonè et al., 2021). Application of intrauterine devices already seems to provide an acceptable approach. Future studies may help to improve the range of available treatments. For individual mares, modification of long‐acting deslorelin formulations to a degree that allows for considerable prolongation of pituitary GnRH receptor down‐regulation might offer a safe, reliable and reversible approach for inhibition of ovarian function. Because of high costs, their usage would probably be limited to valuable mares.

## AUTHOR CONTRIBUTIONS

Both authors contributed equally to the manuscript.

## CONFLICT OF INTEREST

The authors declare that they have no competing interests.
